# Effect of Change of Position (Supine vs. Steep Trendelenburg) on Bispectral Index Value During Robotic Surgery

**DOI:** 10.7759/cureus.29180

**Published:** 2022-09-15

**Authors:** Sachin Kumar, Rohit Balyan, Arjun Nair, Rajesh Tope, Vijay Kumar, Tulika Shrivastava, Anshul ., Rajni Kalia, Jasleen Kaur

**Affiliations:** 1 Anesthesiology, All India Institute of Medical Sciences, New Delhi, IND; 2 Anaesthesiology, Maulana Azad Medical College, Delhi, IND; 3 Anaesthesiology, Indraprastha Apollo Hospitals, Delhi, IND; 4 Anaesthesiology, Pandit Bhagwat Dayal Sharma Post Graduate Institute of Medical Sciences, Rohtak, IND; 5 Anaesthesiology, Government Medical College & Hospital, Chandigarh, IND; 6 Anaesthesiology, Maharishi Markandeshwar University (MMU) Kumarhatti, Solan, IND

**Keywords:** bis, robotic hysterectomies, robotic prostatectomies, robotic surgery, depth of anaesthesia, steep trendelenburg, bispectral index

## Abstract

Background: Bispectral Index (BIS) is used to monitor anesthetic depth and is a useful instrument to keep a check on intraoperative awareness. But there are various situations in which it shows false readings. Our aim of the study was to observe the changes in BIS value with steep Trendelenburg position, which is usually done, in robotic pelvic surgeries.

Methods: We included 100 patients in our study who underwent robotic prostatectomies and hysterectomies. After anesthetizing the patient, the patient's heart rate, systolic blood pressure, mean arterial pressure, end-tidal desflurane, end-tidal CO_2_, and BISwere recorded at three min. intervals, for 15 minutes before and 15 minutes after the Trendelenburg position without surgical stimulus.

Results: We found a significant increase in BIS values (p <0.05) after the change of position from supine to steep Trendelenburg. Heart rate, systolic blood pressure, and mean arterial pressure were almost constant.

Conclusion: Our study concluded that the BIS value increases when patients were shifted from the supine to Trendelenburg position, which might raise the concern of a decrease in anesthetic depth.

## Introduction

In modern clinical practice achieving an adequate depth of anesthesia is desirable. Therefore, monitoring and assessment of the depth of anesthesia are required. Changes in certain clinical signs like blood pressure, pulse rate, the diameter of pupils, sweating, tears, and end-tidal inhalational agent concentration are commonly used to ensure adequate analgesia and depth of anesthesia [1]. These days monitoring of electrical activity of the brain is also used to keep track of the depth of anesthesia. One of these monitoring devices is the Bispectral index (BIS) [2].

BIS monitor was introduced by Aspect Medical Systems to measure unconsciousness levels. BIS uses the data derived from electroencephalogram (EEG) to calculate the hypnotic component of an anesthetic agent. BIS values are unitless and range from 0 to 100. A hundred numerical value shows a fully awake state, and zero indicates an isoelectric state. To ensure adequate anesthetic depth, a BIS value between 40-60 is required, as proved by many investigators [3]. Despite BIS being a useful instrument to keep a check on the anesthetic depth, it has some shortcomings. BIS monitor shows a paradoxical increase with ketamine use, electrocautery, and with atrial pacing [4,5]. Investigators have found that BIS value decreases in certain neurological conditions like cerebral palsy, Alzheimer's, and severe brain injury [6-8]. BIS value also decreases with neuromuscular blocker use in hypothermia and hypoglycemia [9,10]. A strong correlation has been found between cerebral perfusion and BIS values [11].

Kaki and Almarakbi found out that BIS values changed when the position of the head was changed to up or down from a supine position [12]. However, the surgical stimulus was present during observations. Later on, Sang Wook Lee et al. did a study in which they compared BIS values in supine vs. sitting positions [13]. They found that the BIS value got decreased significantly when the position was changed from supine to sitting. Robotic pelvic surgeries require a steep Trendelenburg position, and prolonged surgery in this position can significantly compromise cerebral hemodynamic homeostasis [14].

Hence, we designed this study to evaluate change in BIS value after steep Trendelenburg position from a supine position, during robotic surgeries when no surgical stimulus was started, and anesthetic depth was kept constant. We also correlated the observed changes in BIS values with changes in clinical parameters used routinely to ensure adequate depth of anesthesia.

## Materials and methods

After hospital ethics committee approval (101-20120-132-109045) and written informed consent from 100 patients, we conducted the study at Indraprastha Apollo Hospitals, Sarita Vihar, New Delhi, on patients posted for robotic pelvic surgery (prostatectomy and hysterectomy). The type of study was a hospital-based prospective, observational, non-interventional study. Patients themselves acted as controls, as changes in parameters with change in position, i.e., supine to Trendelenburg position, were seen.

Patients of ASA classes 1 and 2, of > 18 years from either sex scheduled for robotic pelvic surgery were included. Patients with a history of neurological disease, a previous history of head injury, glaucoma, retinal detachment, and patients with uncontrolled hypertension were excluded. Patients with a history of allergy or contraindication to any anesthetic drug were also excluded from the study.

The pre-anesthetic regimen and anesthesia procedure were kept standard for all patients. The procedure for BIS application and advantages of BIS monitoring was explained to every patient, and a patient information sheet was given to the participant to read. Informed consent for anesthesia and BIS monitoring was taken. American Society of Anaesthesiologists (ASA) Task Force guidelines for pre-operative fasting were carried out. After transfer to the operating room, baseline parameters, including electrocardiography (ECG), systolic blood pressure (SBP), mean arterial pressure (MAP), oxygen saturation (SpO2), heart rate, and temperature were recorded. After alcohol cleaning, a disposable BIS electrode was applied to the patient’s forehead as per standard protocol. The patient was pre-oxygenated with 100% oxygen for 3 minutes, and induction of anesthesia was done with an injection of Fentanyl 1.5mcg/kg intravenous (IV), propofol 2.5mg/kg IV. Endotracheal intubation was facilitated by injection of atracurium 0.5mg/kg IV. Desflurane was used for maintenance of anesthesia and after attainment of the constant end-tidal inhalational agent concentration patient was maintained on the low fresh gas flow of 500ml/min of oxygen and air. In all the patients, a tidal volume of 7 ml/kg was used, and respiratory rate was kept between 12-14 minutes to maintain end-tidal carbon dioxide (CO2) within normal limits. Morphine 0.1mg/kg was given at the time of incision, and relaxation was maintained with atracurium infusion @0.5mg/kg/hr.

Intraoperative monitoring involved ECG, HR, invasive arterial blood pressure (SBP, MAP), BIS, end-tidal inhalational agent concentration, EtCO2, SpO2, intraoperative sweating, and tears.

Pre-induction values of all the parameters were recorded, and surgeons created pneumoperitoneum after induction. All the parameters were monitored till the Trendelenburg position was made at an interval of 3 minutes for 15 minutes. (We took readings of all parameters at an interval of 3 minutes till Trendelenburg positioning, but for calculation purposes, we recorded 3 min. interval readings of the last 15 minutes before Trendelenburg.) After Trendelenburg, also all the parameters were noted at an interval of 3 minutes for 15 minutes.

The end-tidal concentration of the inhalational agent was kept the same for 15 minutes before and 15 minutes after the Trendelenburg position, and surgeons were asked not to stimulate the patient during this period. After completion of the surgery, residual neuromuscular blockade was reversed with an injection of glycopyrrolate 0.01 mg/kg and neostigmine 0.05mg/kg. After checking signs of adequate reversal, the patient was extubated and transferred to the post-anesthesia care unit (PACU).

Assessment of change in bispectral index value due to change in position during robotic surgeries when no surgical stimulus was started was one of the outcome measures, and whether this change in bispectral index value was associated with changes in clinical parameters used to monitor the depth of anesthesia was also assessed.

## Results

**Figure 1:**
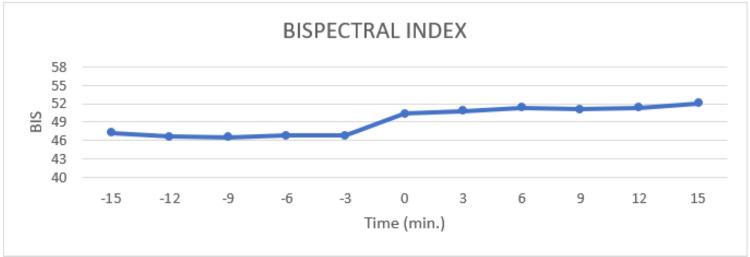
Bispectral index trend at the 3-minute interval before and after Trendelenburg position. 0 min. shows value just after Trendelenburg's position

A total of 100 patients were enrolled in our study, out of which 38 underwent robotic radical hysterectomies, and 62 underwent robotic radical prostatectomies. 38% of the patients were females, and 62% were males. The mean body mass index (BMI) of the patients was 27.0 ± 3.8. Patients themselves acted as control as changes in parameters with respect to change in position, i.e., from supine to Trendelenburg position, were observed. There was a significant increase in BIS values after the Trendelenburg position (p-value <0.05) (Figure 1).

SBP and MAP also increased, but this was not found to be significant. The average of each parameter for each patient was taken (Table 1) 15 minutes prior and 15 minutes after Trendelenburg, i.e., (15, 12, 9, 6, 3 minutes values before Trendelenburg vs. just after, 3, 6, 9, 12 minutes after Trendelenburg) and then these averages were compared. There was a significant increase in BIS values after the Trendelenburg position. We took the mean of all the parameters at different time intervals (Table 2) and compared pre-Trendelenburg values of all the parameters with post-Trendelenburg 0 min., 3, 6, 9, 12, and 15 min. values.

**Table 1:** Comparison of parameters 15 minutes before and after Trendelenburg position HR: Heart rate, SBP: Systolic blood pressure, MAP: Mean arterial pressure, BIS: Bispectral index, EtDES: End-tidal desflurane

	Before Trendelenburg	After Trendelenburg	
	Mean ± SD		P value
HR	70.82±9.33	70.36±9.73	0.123
SBP	120.88±12.24	121.23±12.25	0.235
MAP	89.93±9.41	90.45±9.14	0.102
BIS	46.83±4.10	51.01±4.18	<0.01
EtDES	6.21±0.33	6.21±0.33	0.963

**Table 2:** -3, 0, 3, 6, 9, 12, 15 Time in minutes. (0 minute is just after Trendelenburg) HR: Heart rate, SBP: Systolic blood pressure, MAP: Mean arterial pressure, BIS: Bispectral index, EtDES: End-tidal desflurane

	-3 min.	0 min.	3 min.	6 min.	9 min.	12 min.	15 min.
	MEAN ± SD						
HR	70.52±9.74	70.65±9.91	70.48±9.91	70.28±9.84	70.19±9.62	70.22±9.74	70.53±9.49
SBP	121.80±12.95	121.59±12.18	121.30±12.23	121.65±12.17	122.35±12.32	121.58±12.44	121.46±12.92
MAP	90.33±9.55	90.17±9.54	90.83±9.51	90.71±9.18	91.10±8.93	90.48±9.27	90.92±9.56
BIS	46.88±4.47	50.47±4.41	50.83±4.45	51.27±4.32	51.17±4.60	51.31±4.19	52.05±4.20
EtDES	6.22±0.33	6.21±0.32	6.21±0.33	6.21±0.33	6.21±0.33	6.22±0.33	6.21±0.32

**Figure 2:**
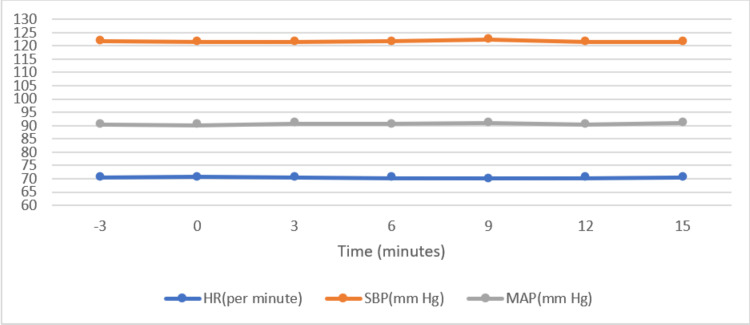
Trends of heart rate (HR), systolic blood pressure (SBP) and mean arterial pressure (MAP)

We found that there was a statistically significant rise in BIS values in all the comparisons with a p-value less than 0.05 (Table 3). There was a slight rise in mean arterial blood pressure and systolic blood pressure value in comparison to just before the Trendelenburg position and at 9 minutes after the Trendelenburg position, but none of these changes were statistically significant (Figure 2).

**Table 3:** P-values for comparison of just before Trendelenburg values with after Trendelenburg values of different parameters. (p value <0.05 taken as significant) HR: Heart rate, SBP: Systolic blood pressure, MAP: Mean arterial pressure, BIS: Bispectral index, EtDES: End-tidal desflurane

	P Value					
	-3 min. vs. 0 min.	-3 min. vs. 3 min.	-3 min. vs. 6 min.	-3 min. vs. 9 min.	-3 min. vs. 12 min.	-3 min. vs. 15 min.
HR	0.515	0.635	0.198	0.116	0.173	0.168
SBP	0.486	0.328	0.662	0.060	1.00	0.368
MAP	0.450	0.071	0.376	0.067	0.346	0.246
BIS	0.008	0.008	0.008	0.007	0.011	0.009
EtDES	0.299	0.148	0.237	0.384	0.631	0.521

We also looked for sweating, tears, and pupillary dilation during our observation period in all patients, but none of them showed these signs. In PACU, upon asking for awareness during anesthesia, none of the patients gave a history of the same.

## Discussion

Steep Trendelenburg position produces significant changes in hemodynamics and cerebral homeostasis, which might alter the depth of anesthesia [15]. The primary objective of this study was to find out the effect of the steep Trendelenburg position on the BIS values in comparison to supine values by keeping end-tidal inhalational agent concentration constant. We also tried to correlate this change by measuring the changes in other clinical parameters. After the creation of the pneumoperitoneum, we requested the surgeon not to proceed with definitive surgery 15 minutes before and after the Trendelenburg position. Interestingly we found a significant rise in bispectral index values (p<0.05) when the patient was placed in a steep Trendelenburg position > 40 degrees, but in all observations recorded BIS values were within acceptable limits. As observed in previous studies, we also did not find any significant change in vital parameters, i.e., heart rate, SBP, and MAP. There was a slight increase in SBP and MAP at 9 minutes compared to the supine value (3 min. before Trendelenburg), which was not significant (p>0.05), and this was in correlation with the study done by Kaki and Almarakbi [12]. These changes can be explained by the changes in stroke volume, cardiac output, and end-diastolic volume [16]. We also looked for sweating and tears during our observation period in all patients, but none of them showed these signs.

Kaki and Almarakbi observed an increase in BIS readings with Trendelenburg position and a decrease in BIS readings with head up position [12]. They associated these changes with the change in cerebral hemodynamics, cerebral blood flow (CBF), intracranial pressure (ICP), and cerebral perfusion pressure (CPP) [17]. They concluded that changes in cerebral blood flow can alter cerebral electrical activity responsible for EEG waveform changes [18]. They did not avoid the influence of surgical stimulus while measuring BIS and MAP values. They also said that the rise in these values can be because of surgical stimulus. This relation of alteration in cerebral electrical activity with respect to the alteration in cerebral blood flow has been confirmed by Buget et al. [19]. By using internal carotid artery and vertebral artery doppler ultrasound, they found that cerebral blood flow got decreased in sitting position, and they also found an associated decrease in the value of patient state index (PSI). Sang Wook Lee et al. compared BIS values in supine vs. sitting positions without surgical stimulus [12]. They found the decrease in value of BIS when the patient's position was changed to sitting from supine and attributed this change to decreased cerebral blood flow. Mallick et al. also found a significant (P<0.05) increase in the value of BIS when they shifted their patients from Trendelenburg position, <30 degrees to more than 30-degree Trendelenburg [20]. In their study, they did not avoid the surgical stimulus.

But we avoided the surgical stimulus during our study by asking the surgeons not to stimulate the patients during our observation period. We also kept the anesthetic depth constant by keeping the end-tidal inhalational agent constant.

There are certain limitations in our study. We could have validated our findings by measuring cerebral blood flow or cerebral perfusion, but unfortunately, our facility lacks monitoring equipment to measure the same in the operation theatre. Also, we could have observed our results separately in ASA grade 1 and ASA grade 2 patients as some hypertensive and diabetics have altered hemodynamics; however, we found a rise in values of the Bispectral index in all patients. Another limitation in our study is that we could have taken readings at the end of the surgery when a balanced steady state was reached, but as the duration of surgeries was different, we were unable to do so.

## Conclusions

In this study, we observed a change in BIS value from 46 to 52 with the change in position. This change was significant but was not associated with any signs of light anesthesia. The rise in BIS values with Trendelenburg position was probably because of position-related effects on cerebral blood flow and cerebral electrical activity. The use of BIS in surgeries involving Trendelenburg positioning might raise a false alarm showing the patient going in a light plane of anesthesia when a position is changed from supine to Trendelenburg. This should be kept in mind while using BIS to monitor the depth of anesthesia for correct interpretation. Further study might be required to clarify the concern and formulate guidelines for ensuring safe anesthesia practice.
